# Prevalence and Risk Factors of Burnout Among Medical Residents in Tunisia: A Cross-Sectional Study

**DOI:** 10.1192/j.eurpsy.2024.1732

**Published:** 2024-08-27

**Authors:** W. Haouari, S. Omri, A. Labyadh, I. Gassara, R. Feki, N. Charfi, J. Ben Thabet, M. B. Maalej, N. Smaoui, L. Zouari, M. Maalej

**Affiliations:** 1 Psychiatry C, Hedi Chaker university hospital, Sfax, Tunisia

## Abstract

**Introduction:**

The burnout syndrome is a blend of physical exhaustion and emotional fatigue that impairs an individual’s performance at work. In Tunisia, factors like working hours, the frequency of monthly shifts, and the physical and emotional abuse that physicians face from patients have collectively led to a significant incidence of burnout among medical professionals.

**Objectives:**

To evaluate the prevalence of burnout syndrome among medical residents working in healthcare facilities in Tunisia and to pinpoint the contributing factors.

**Methods:**

This study is a descriptive cross-sectional survey conducted among medical residents completing their training in various healthcare facilities in Tunisia. The study employed an online self-administered questionnaire and assessed burnout across three dimensions: personal burnout, professional burnout, and relational burnout, using the Copenhagen Burnout Inventory (CBI).

**Results:**

A total of 50 physicians took part in the survey. Among them, 72% were female, 80% were single, and the average age at the time of the study was 27.72 years. Concerning their professional status, 84% worked in university hospitals, 16% specialized in surgery, 40% specialized in medicine, and 44% were family physicians. The majority were students from the Faculty of Medicine in Sfax (56%), with 30% in Monastir, 8% in Tunis, and 6% in Sousse. Regarding their work hours, more than 40 hours per week were reported by 32% of participants. According to the CBI scale, 12% of participants had scores indicating severe personal burnout, while 20% had scores indicating moderate personal burnout. Additionally,16% reported severe professional burnout, and 12% had scores suggesting severe relational burnout. In contrast, only 8% had scores indicating moderate relational burnout. The sociodemographic and professional factors that were studied, such as weekly working hours, monthly shifts, specialty, and workplace, did not show a significant correlation with the presence of burnout syndrome.

**Conclusions:**

Burnout syndrome among medical resident physicians not only impacts their physical and mental well-being but also reduces their effectiveness and motivation at work. It is essential to introduce stress management strategies within hospitals to foster a healthier work-life balance.

**Disclosure of Interest:**

None Declared

